# *CACNA1H* Calcium Channel Mutations in Primary Aldosteronism – Is Sodium the Culprit?

**DOI:** 10.1210/endocr/bqaa173

**Published:** 2020-11-03

**Authors:** Gabriel Stölting, Ute I Scholl

**Affiliations:** 1 Charité – Universitätsmedizin Berlin, Corporate Member of Freie Universität Berlin, Humboldt-Universität zu Berlin, and Berlin Institute of Health, Department of Nephrology and Medical Intensive Care, Berlin, Germany; 2 Berlin Institute of Health (BIH), Berlin, Germany; 3 Charité – Universitätsmedizin Berlin, Corporate Member of Freie Universität Berlin, Humboldt-Universität zu Berlin, and Berlin Institute of Health, BIH Center for Regenerative Therapies, Berlin, Germany

**Keywords:** aldosterone, electrophysiology, calcium imaging, CaV3.2

Primary aldosteronism (PA), the most common cause of secondary hypertension, is characterized by an excessive production of the steroid hormone aldosterone relative to blood potassium and the circulating levels of renin, which normally jointly (directly or indirectly) control its synthesis. PA is frequently caused by either bilateral hyperaldosteronism (bilateral adrenal hyperplasia) or aldosterone-producing adenomas. In studies performed over the past decade, PA has been shown to be largely genetic in etiology. Somatic mutations in ion channels or pumps account for the majority of aldosterone-producing adenomas and are also found in bilateral hyperaldosteronism. In addition, rare patients with familial hyperaldosteronism (FH) carry germline mutations, often in the same genes. The pathophysiology of such mutations is typically based on increased intracellular calcium signaling, which governs expression of aldosterone synthase. This can occur directly, when mutant calcium channels allow for increased calcium influx, or indirectly. An example of an indirect mechanism are mutations in the KCNJ5 potassium channel that allow for abnormal sodium permeability and influx, cellular depolarization, and subsequent activation of voltage-gated calcium channels (1).

The T-type voltage-gated calcium channel CACNA1H (Ca_V_3.2) has been linked to membrane potential oscillations of the murine zona glomerulosa and aldosterone production (2). Mutations in *CACNA1H* cause FH-IV when present in the germline (3) and are rarely found as somatic mutations in aldosterone-producing adenomas (4). The initial electrophysiological analysis of the most common mutation in Ca_V_3.2, M1549V, when expressed in HEK293 cells, suggested a direct gain of calcium channel function, with impaired inactivation and a small shift of activation toward more hyperpolarized potentials (3).

In this issue, the group of Sascha Bandulik expands on this work and discovers an unanticipated role of sodium permeability in the pathophysiology of FH-IV, suggesting an additional indirect mechanism of increased calcium signaling (5). The use of the H295R adrenocortical cell line and of solutions with a more physiological calcium concentration than in prior studies allows them to correlate the changes in Ca_V_3.2 function to membrane depolarization and calcium dynamics in a more native environment. Under these conditions, the shift of the activation curve of mutant channels leads to the activation of mutant channels at or very close to the resting potential of around –80 mV, which explains autonomous aldosterone production. Interestingly, the activation of both wild-type and mutant Ca_V_3.2 appears to be shifted to more hyperpolarized potentials in H295R cells compared with recordings from nonadrenal Chinese hamster ovary cells. It would be interesting to further investigate the underlying mechanism. Could endogenously expressed proteins, such as calcium channel subunits, posttranslational modifications, or other factors in adrenal cells cause a shift in the voltage dependence of activation? In any case, pronounced voltage oscillations (although at lower frequency than those seen in rodent adrenal slices (2)) can be induced in cells transfected with mutant (but rarely wild-type) channels, and cytosolic calcium concentrations are elevated compared to cells transfected with wild-type (5). Somewhat divergent phenotypes of individual cells may be due to the fact that H295R cells are not clonal in origin and that transient transfections were used.

The pathophysiologic role of sodium permeability may be even more interesting. Voltage-gated calcium channels share a common ancestor with other cation channels such as sodium and potassium channels, and it has been suggested that some voltage-activated calcium channels may still exhibit a significant permeability for sodium ions (6). It appears from the study by Gürtler et al that, at least in H295R cells, the mutant Ca_V_3.2 M1549V channel has a higher sodium permeability than wild-type channels. Removal of extracellular sodium attenuated calcium influx into cells transfected with mutant channels, suggesting a role of the sodium conductance in the pathophysiology of the disease. Interestingly, another voltage-gated calcium channel mutated in PA, the L-type channel Ca_V_1.3, has been suggested to also harbor a significant sodium permeability aiding in the depolarization of sinoatrial node cells (7). It will require further biophysical analyses to see whether and, if so, how an increased sodium permeability of Ca_V_3.2 (and perhaps also Ca_V_1.3) may contribute to electrical signaling given that the driving force for calcium ions is much higher because of its larger concentration gradient. Also, it would be of interest to study other mutations in Ca_V_3.2 linked to PA to see if the increased sodium permeability is a common trait, especially given that the phenotypes of some of the reported mutations were distinct from those of M1549V (8).

Clearly, Gürtler et al have elegantly confirmed earlier hypotheses that mutant Ca_V_3.2 results in higher calcium influx using a model system that is much closer to zona glomerulosa than previously used systems. It will be interesting to study more closely the effects of sodium permeability in even more native contexts such as knock-in animal models or human samples where other regulatory mechanisms might further affect the phenotype of these mutations.

## Data Availability

Data sharing is not applicable to this article as no datasets were generated or analyzed during the current study.

## Additional Information

***Disclosure Summary:*** U.I.S. receives funding from the Stiftung Charité (BIH_PRO_406) and the Deutsche Forschungsgemeinschaft (SFB 1365). G.S. receives funding from the Deutsche Forschungsgemeinschaft (STO 1260/1-1).
